# Supplementary material for: Manipulation of emergent vortices in swarms of magnetic rollers
Source: Nat Commun. 2018 Jun 14;9:2344. doi: 10.1038/s41467-018-04765-w (PMC6002404; doi:10.1038/s41467-018-04765-w)
Supplement: Supplementary file 1 — Supplementary Information [file 41467_2018_4765_MOESM1_ESM.pdf]

Supplementary Information for Manipulation of emergent vortices in swarms of magnetic rollers

Gašper Kokot and Alexey Snezhko

Materials Science Division, Argonne National Laboratory, 9700 South Cass Avenue, Argonne, IL 60439, USA

Corresponding author, snezhko@anl.gov

May 14, 2018

Supplementary Figures

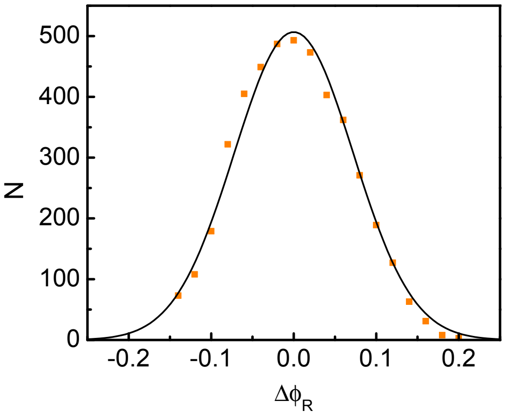

Supplementary Figure 1: Order parameter fluctuations. The probability histogram ($\sum N = 4446$) of order parameter fluctuations $\Delta\phi_{\mathrm{R}}$ displays a normal distribution (the black line is a Gaussian fit).

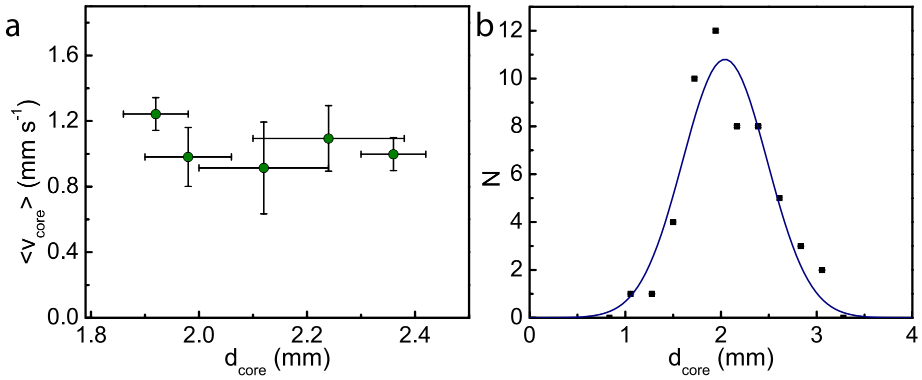

Supplementary Figure 2: Vortex on a flat surface. **a**, Mean vortex core speed versus roller vortex core diameter. Error bars are the standard deviation of the measurements. **b**, The histogram ($\sum N = 54$) of vortex core diameters d (the blue line is a Gaussian fit) realised on a flat surface.

Supplementary Notes

Supplementary Note 1

The vortex order parameter ϕ_{R} (see main text) fluctuates around a mean value. To characterize these fluctuations we calculate the deviation from the mean value $\Delta\phi_{R_i} = \phi_{R_i} - \frac{1}{N}\sum_{i=1}^{N}\phi_{R_i}$, where i runs over all N vortex realizations. Figure 1 demonstrates the Gaussian distribution of $\Delta\phi_{\mathrm{R}}$. The experiment was carried out in a spherical potential well (radius of curvature 52 mm) with Ni particles (radius $R_{\mathrm{Ni}} = 69\,\mu\mathrm{m}$) subjected to in- and out-of-plane oscillating magnetic field (amplitude $B_0 = 5.78\,\mathrm{mT}$, frequency $f_{\mathrm{B}} = 40\,\mathrm{Hz}$).

Supplementary Note 2

For vortices on a flat surface far from container walls (no confinement) the mean vortex core speed $\langle v_{\mathrm{core}}\rangle$ does not depend on the vortex core diameter d_{core} (Figure 2a), as determined from the peak of radially averaged tangetial velocity (see Figure 1d). The vortex core diameters d_{core} display a wide distribution (Figure 2b). The experiment was carried with Ni particles (radius $R_{\mathrm{Ni}} = 69\,\mu\mathrm{m}$) subjected to in- and out-of-plane oscillating magnetic field (amplitude $B_0 = 5.66\,\mathrm{mT}$, frequency $f_{\mathrm{B}} = 42\,\mathrm{Hz}$).
