# Supplementary material for: Manipulation of emergent vortices in swarms of magnetic rollers
Source: Nat Commun. 2018 Jun 14;9:2344. doi: 10.1038/s41467-018-04765-w (PMC6002404; doi:10.1038/s41467-018-04765-w)
Supplement: Supplementary file 3 — Description of Additional Supplementary Files [file 41467_2018_4765_MOESM3_ESM.pdf]

Description of Additional Supplementary Files

File Name: Supplementary Movie 1
Description: Emergent roller vortex in soft harmonic gravitational confinement. frequency is 40 Hz, field amplitude is 6mT.

File Name: Supplementary Movie 2
Description: Roller vortex. Slow motion (Playback is a half of the real time). Soft harmonic gravitational confinement.

File Name: Supplementary Movie 3
Description: Roller vortex at a flat surface. 42Hz, 7mT.

File Name: Supplementary Movie 4
Description: Spontaneous chirality switching event for the roller vortex selfassembled at 40Hz, 6 mT field.

File Name: Supplementary Movie 5
Description: Rollers at a flat surface in air. 42Hz, 7mT.

File Name: Supplementary Movie 6
Description: Bead trapping and transport by a roller vortex at a flat surface. 42Hz, 7mT.
